# Demonstration of simultaneous experiments using thin crystal multiplexing at the Linac Coherent Light Source

**DOI:** 10.1107/S1600577515003999

**Published:** 2015-04-10

**Authors:** Y. Feng, R. Alonso-Mori, T. R. M. Barends, V. D. Blank, S. Botha, M. Chollet, D. S. Damiani, R. B. Doak, J. M. Glownia, J. M. Koglin, H. T. Lemke, M. Messerschmidt, K. Nass, S. Nelson, I. Schlichting, R. L. Shoeman, Yu. V. Shvyd’ko, M. Sikorski, S. Song, S. Stoupin, S. Terentyev, G. J. Williams, D. Zhu, A. Robert, S. Boutet

**Affiliations:** a Linac Coherent Light Source, SLAC National Accelerator Laboratory, 2575 Sand Hill Road, Menlo Park, CA 94025, USA; b Max-Planck Institute for Medical Research, Heidelberg, Germany; c Technological Institute for Superhard and Novel Carbon Materials, Troitsk, Russia; d Advanced Photon Source, Argonne National Laboratory, 9700 South Cass Avenue, Argonne, IL 60439, USA

**Keywords:** FEL, X-ray, multiplexing, diamond, thin-crystal

## Abstract

Multiplexing of the Linac Coherent Light Source beam was demonstrated for hard X-rays by spectral division using a near-perfect diamond thin-crystal monochromator operating in the Bragg geometry. The wavefront and coherence properties of both the reflected and transmitted beams were well preserved, thus allowing simultaneous measurements at two separate instruments. In this report, the structure determination of a prototypical protein was performed using serial femtosecond crystallography simultaneously with a femtosecond time-resolved XANES studies of photoexcited spin transition dynamics in an iron spin-crossover system. The results of both experiments using the multiplexed beams are similar to those obtained separately, using a dedicated beam, with no significant differences in quality.

## Abstract 2

A demonstration of multiplexing the LCLS beam by spectral division using a near-perfect diamond thin-crystal monochromator is presented.

## 1. Introduction   

The advent of X-ray free-electron lasers (FELs) such as the Linac Coherent Light Source (LCLS) (Emma *et al.*, 2010 ▶) in the USA and the SPring-8 Angstrom Compact Free Electron Laser (SACLA) (Ishikawa *et al.*, 2012 ▶) in Japan has generated tremendous excitement among the X-ray user community because of the enormous potential of such sources for transforming scientific research in a wide range of disciplines, including physics, chemistry, biology, material science and energy research. Since the start of user operation of LCLS in 2009 and SACLA in 2011, experiments at these two user facilities have already made many significant findings and breakthroughs (Young *et al.*, 2010 ▶; Chapman *et al.*, 2011 ▶; Seibert *et al.*, 2011 ▶; Boutet *et al.*, 2012 ▶; Glover *et al.*, 2012 ▶; Ringer *et al.*, 2012 ▶; Milathianaki *et al.*, 2013 ▶; Trigo *et al.*, 2013 ▶). As a result, the demand for user access has steadily increased, leading to approximately only one out of five user proposals ultimately being granted beam time at LCLS. This shortfall stems from the serial nature of X-ray FEL operation whereby a single undulator (Emma *et al.*, 2010 ▶) typically can only provide the beam to one experimental station at any given time, in strong contrast to the scenario at storage-ring synchrotron X-ray sources where many tens of instruments can receive X-ray beams concurrently.

Much effort has been devoted at LCLS to splitting the beam to service multiple endstations, creating additional beam time capacity for users (Feng *et al.*, 2012*b* ▶, 2013*a* ▶,*b* ▶). By taking advantage of a sufficiently broad natural bandwidth [of the order 3 × 10^−3^ (Zhu *et al.*, 2012 ▶)] generated *via* the self-amplified spontaneous emission (SASE) process (Kondratenko & Saldin, 1979 ▶; Bonifacio *et al.*, 1984 ▶), a successful implementation of spectral division for hard X-rays using a thin single-crystal monochromator has recently been reported (Zhu *et al.*, 2014 ▶). The monochromator crystal used was a 105 µm-thick type IIa diamond single-crystal working in the Bragg geometry, producing two well separate beams: one Bragg reflected and monochromatic, the other in transmission with a spectral notch created by the Bragg reflection. The beam profiles of the transmitted beam were measured with and without the diamond crystal inserted, and no discernible qualitative differences were found, suggesting that its wavefront and coherence properties were relatively well preserved. For the reflected beam, possible wavefront distortions were investigated by studying its focusing characteristics. When compared with monochromatic beam produced using thick Si crystals, an additional but small angular divergence was found in the diffraction plane. The ultimate validation of the performance of this multiplexing scheme, however, can only be made from experimental evidences using real systems.

In this article we present the findings from performing simultaneous measurements on two benchmarking systems, *i.e.* serial femtosecond crystallography of the prototypical protein lyzozyme using the transmitted beam; and photoexcited spin transition dynamics studies of an iron spin-crossover system, iron(II) tris(2,2′-bipyridine) in water *via* femtosecond time-resolved X-ray Absorption Near-Edge Spectroscopy (XANES) measurement using the Bragg-reflected beam.

## 2. Experimental setups and methods   

**Figure 1:**
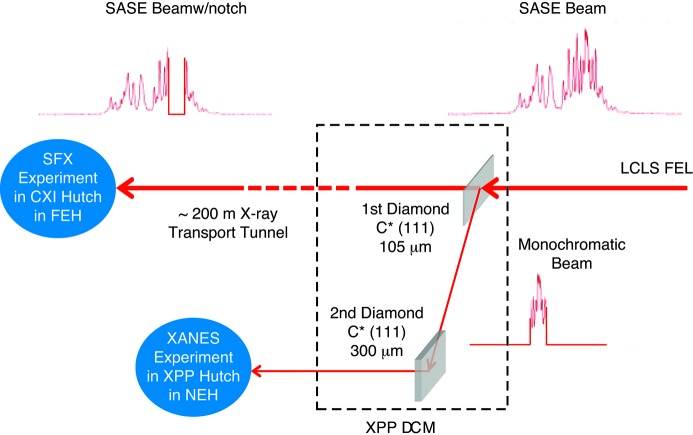
Schematics of the XPP–CXI spectral division multiplexing scenario involving the XPP and CXI instruments for two simultaneous experiments using the transmitted and reflected beams generated from a ∼105 µm diamond thin-crystal diffracting in the Bragg geometry. The spectral content of the transmitted beam is essentially that of the incident SASE (pink) beam with a notch created by the Bragg reflection, whereas the bandwidth of the reflected (monochromatic) beam is much narrower, containing only a few spectral spikes.

The experiments were performed using one of the many possible multiplexing scenarios involving two of the four LCLS hard X-ray instruments (Feng *et al.*, 2013*a* ▶), *i.e.* the X-ray Pump and Probe (XPP) instrument (Chollet *et al.*, 2015 ▶) in the Near Experimental Hall (NEH) and the Coherent X-ray Instrument (CXI) (Liang *et al.*, 2015 ▶) in the Far Experimental Hall (FEH) as shown schematically in Fig. 1 ▶. The spectral division beam-splitting diamond thin-crystal (first crystal) was installed in the large-offset Double-Crystal Monochromator (DCM) of the XPP instrument. The transmitted beam continued to propagate ∼200 m downstream to the CXI endstation for conducting the protein crystallography measurements, while the reflected monochromatic beam was again Bragg reflected by a second thick diamond crystal and remained in the XPP instrument for conducting the time-resolved XANES studies.

### 2.1. Beam multiplexing using spectral division   

There are many different techniques for splitting an X-ray beam. Wavefront division has been demonstrated for soft X-ray FELs using mirrors (Sorgenfrei *et al.*, 2010 ▶); but for hard X-rays it would require long optics which would occupy a large footprint due to the small critical angle for total external reflection.

Amplitude division typically used for splitting optical beams is very challenging for X-rays again because of the small critical angle and large absorption coefficient. However, spectral division using thin-crystal monochromators represents a viable technique and has long been used by the synchrotron X-ray user community for beam multiplexing (Als-Nielsen *et al.*, 1994 ▶; Grübel *et al.*, 1994 ▶, 1996 ▶; Tsusaka *et al.*, 2001 ▶). It has also been applied to build sophisticated FEL optical devices such as the split-and-delay unit (Roseker *et al.*, 2009 ▶; Stetsko *et al.*, 2013 ▶) with the aim of performing ultrafast X-ray photon correlation spectroscopy (XPCS) experiments (Grübel *et al.*, 2008 ▶).

At typical hard X-ray energies, this technique offers the advantage that a relatively large beam separation can be achieved due to the high diffraction angle, and the device size can be made relatively compact. The most challenging aspect, however, lies in the diffraction quality of the thin crystal which must be maintained while the sample is being irradiated by highly brilliant X-ray FEL beams.

#### 2.1.1. Optics using diamond thin-crystals   

The most perfect crystals available for hard X-ray monochromators are silicon single crystals. Not only do they offer exceptionally high diffraction quality but they are also ubiquitous and come in practically any size and orientations. These scientific benefits are the fruits reaped from decades of technological advances and innovations by the semiconductor industry, especially in the areas of crystal growth and polishing techniques. As such, Si single crystals are widely used by the synchrotron and X-ray FEL communities alike for thick-crystal monochromators and mirrors or mirror substrates.

A relatively small thickness is needed when using Si crystals for spectral division of X-ray beams. For example, at a typical X-ray energy of 8 keV using a 〈111〉 symmetrically cut thin crystal in the Bragg diffraction geometry, achieving 50% beam transmission would require thinning the crystal down to approximately 12 µm. Manufacturing such a thin specimen while maintaining lattice perfection and having it mounted free of strain can be very challenging despite much concerted effort by many (Feng *et al.*, 2012*b* ▶; Osaka *et al.*, 2012 ▶). More importantly, it was observed that a thin Si membrane sample would exhibit significant vibrations and thus local bending when exposed to an unattenuated X-ray FEL beam at a typical energy of a few mJ pulse^−1^ (Feng *et al.*, 2012*b* ▶, 2013*b* ▶). These vibrations were likely induced by thermal-acoustic shock waves which were generated by the almost instantaneous energy deposition from photoabsorption of the ultra-short (typically a few to 50 fs) FEL pulses. The vibrations were very long lived with a time constant of order tens to hundreds of milliseconds, rendering the crystal completely unusable as a FEL beam splitter if operating at a repetition rate of greater than 1 Hz. The long lifetime was ironically attributed to the perfection of the Si lattice, making the crystal membrane an almost ideal high *Q*-factor oscillator.

Diamond single crystals, on the other hand, are not only much stiffer mechanically but also allow the use of thicker samples because of the smaller photoabsorption coefficient. At 8 keV using a 〈111〉 symmetrically cut thin crystal in the Bragg diffraction geometry, the thickness can be as high as 170 µm while still presenting a transmission higher than 50%. Since a thin plate’s stiffness scales as its thickness cubed and linearly with the Young’s modulus, potential static deformations due to mounting in a thin diamond crystal are reduced by more than four orders of magnitude compared with a much thinner Si crystal (with an identical transmission efficiency). More importantly, dynamic effects such as the vibrations observed in Si are expected to be reduced considerably in diamond due to its relatively higher thermal conductivity and lower thermal expansion coefficient. As such, the use of diamond would always be preferred, were it not for the fact that high-quality thin diamonds of sufficient size with a lattice orientation other than 〈100〉 growth direction have historically been extremely difficult to acquire. Only recently did such diamond samples become more available because of significant progress in diamond growth and polishing techniques (Blank *et al.*, 2007 ▶; Burns *et al.*, 2009 ▶; Shvyd’ko *et al.*, 2010 ▶, 2011 ▶; Polyakov *et al.*, 2011 ▶; Sumiya & Tamasaku, 2012 ▶; Stoupin *et al.*, 2013 ▶).

For the current measurements, a pair of high-quality type IIa 〈111〉 diamond crystals (Stoupin *et al.*, 2014 ▶) was used. They were grown, diced and polished by the Technological Institute for Superhard and Novel Carbon Materials (TISNCM) in Troitsk, Russia, using the temperature-gradient method at high static pressure and high temperatures (Blank *et al.*, 2007 ▶). They were commissioned in the XPP DCM (Zhu *et al.*, 2014 ▶). The two crystals measured roughly 5 mm × 5 mm in size. The first crystal had a 105 µm average thickness with a 5% uniformity. The second diamond crystal was 300 µm-thick. The latter re-directs the monochromatic beam reflected from the first crystal onto an axis parallel but offset to the original beam direction as depicted in Fig. 1 ▶. The 〈111〉 surface of the crystals were intentionally miscut by 2° for easing polishing.

In an earlier *in situ* test, a pair of similar quality diamond crystals also produced by TISNCM were mounted in a miniature silver-plated copper holder using InGa eutectic for making thermal contact without clamping (Feng *et al.*, 2013*a* ▶). The drawback of such a scheme was that the crystals were not sufficiently constrained mechanically, resulting in walking of the reflections off the Bragg condition in an unpredictable manner. This effect is especially significant because of the high degree of collimation for hard X-ray FEL beams which is of the order of a few microradians. Here, both crystals were instead mounted on Chemical-Vapor-Deposition (CVD) diamond miniature frames, and held down by CVD diamond fingers (clips) under optimal tension (Stoupin *et al.*, 2014 ▶). Extensive topographical measurements were carried out before and after mounting to minimize additional lattice strain (Stoupin *et al.*, 2014 ▶). A small but finite temperature rise of 2 K was observed using an infrared camera in the 105 µm crystal when irradiated by the LCLS beam at full power and maximum repetition rate, suggesting that the thermal conduction through the CVD diamond holder was sufficient.

#### 2.1.2. Performances of reflected and transmitted beams   

Beam characterization measurements were preformed at multiple X-ray energies between 7 and 9.5 keV. The LCLS FEL was operated in the standard SASE configuration using an electron bunch charge of 150 pC with optimal compression, producing on average 2 to 3 mJ pulse^−1^. The X-rays were linearly polarized in the horizontal direction. The number of X-ray photons incident onto the first diamond (splitting) crystal was thus estimated to be approximately 1 × 10^12^ per pulse. The wavefront and coherence properties of the two split beams and other multiplexing characteristics such as reflectivity and throughput were then studied and the findings have been described elsewhere (Zhu *et al.*, 2014 ▶) and are summarized below.

*Spectral notch and the Darwin curve.* The spectral content of both the transmitted and reflected beams were measured using hard X-ray high-resolution single-shot spectrometers (Zhu *et al.*, 2012 ▶; Feng *et al.*, 2012*a* ▶). In the transmitted spectra, a notch created by the Bragg reflection was indeed observed after averaging over many pulses. The width of the notch was very close to that of the Darwin width of an ideal diamond 〈111〉 reflection, and so was the spectral width of the reflected beam, suggesting a very high degree of crystalline quality.

*Transmittance and reflectivity.* Both transmittance and the reflectivity were measured at a few X-ray photon energies to within 10% of the calculated values, further confirming that the diffraction quality of the thin diamond sample was excellent. The intensity reduction of the transmitted beam is primarily due to the photoabsorption in the diamond. A small fraction of the loss comes from the reflection of the incident X-rays with energies within the Darwin curve.

*Transmitted beam profile.* A simple approach was first used to characterize the transmitted beam by imaging its beam profile, with and without the diamond inserted. The imaging device was located at the largest possible distance of ∼200 m downstream of the beam-splitting diamond crystal for greater sensitivity. The intrinsic pointing- and positional jitter of the FEL produced smearing effects on the profile, resulting in an effective angular resolution of 0.15 µrad. The uncertainty in the intensity measurement at each point on the profile was completely determined by the noise in the imaging device to be less than 1%. As such, no additional features of greater than 1% in intensity and 0.15 µrad in angular spread were observed with the diamond inserted, suggesting that the wavefront and coherence properties of the transmitted beam were well preserved.

*Transmitted beam angular shift.* A 50 µm lateral shift of the profile centroid was, however, observed 200 m downstream when the diamond crystal was inserted. This shift was attributed to the fact that the two surfaces of the 105 µm crystal were not perfectly parallel. This wedge effectively steered the FEL beam angularly by ∼0.25 µrad, consistent with a wedge angle of 0.1° estimated using optical measurements. A constant translational shift associated with the finite thickness of the diamond sample was calculated to the order of a few nanometres, thus deemed negligible.

*Reflected beam focusing properties.* For the monochromatic reflected beam, the average beam profiles were also imaged but at a much smaller distance of ∼5 m from the splitting crystal. Unlike the Si thin-crystals studied previously (Feng *et al.*, 2012*b* ▶, 2013*b* ▶), here the profile resembled that of incident SASE beam and showed no apparent FEL induced vibrations of greater than 1 µrad in effective angular steering, which is smaller than the natural angular divergence of ∼2.5 µrad for hard X-rays at 8 keV. Static distortion or bending of the thin diamond was evaluated by measuring its focusing characteristics. When compared with that of an un-split beam, an additional angular divergence was found in the diffraction plane, suggesting that residual strain from mounting caused the reflected beam to fan out more and effectively moved the FEL virtual source point downstream by an appreciable amount. The increased beam divergence did not impact the particular XPP pump–probe measurement reported here, but, for future experiments dependent on the X-ray focusing properties, further reduction or compensation of the induced static bending may be required.

### 2.2. Simultaneous measurements on real systems   

The properties of both the transmitted and reflected beams generated from a thin diamond crystal were shown to be similar to those obtained from an ideal crystal, lending credence to the usability for beam multiplexing. The validation, however, must ultimately come from conducting measurements on real experimental systems. The XPP–CXI multiplexing scenario shown in Fig. 1 ▶ was chosen as the first demonstration experiment by pairing the XPP instrument with the CXI instrument running a protein crystallography measurement.

A photon energy of 7.125 keV was chosen as mutually agreed upon by both simultaneous experiments and was primarily driven by the desire to perform XANES measurements at the iron (Fe) *K*-edge at XPP. The average pulse energy from the source was approximately 2 mJ as measured by the gas detector in the LCLS Front-End Enclosure (Hau-Riege *et al.*, 2010 ▶). Using attenuators local to the separate experiments, the maximum FEL fluence on the samples were controlled independently. The repetition rate of the FEL was 120 Hz for maximum data acquisition efficiency. If necessary, the repetition rate of the FEL at each separate experiment could also be varied independently using a local device called a pulse-picker.

A total of five standard LCLS 12 h shifts were allocated to the simultaneous demonstration, as opposed to a total of ten shifts (five plus five) for the first two original individual experiments. Similar data volumes and quality were obtained for both experiments to the original. For the CXI experiment, the beam intensity loss by the insertion of the diamond crystal is minimal and of the order of the SASE FEL intensity fluctuations, and the set-up and data acquisition time were similar to the original measurement. The additional time overhead required by inserting the diamond crystal was of the order of 10 min and thus was negligible. For the XPP experiment, a reduction in the incident X-ray flux required longer data acquisition time than originally as was discussed in §2.2.2, and the additional alignment overhead of the diamond crystal was also negligible.

#### 2.2.1. Serial femtosecond crystallography at CXI   

The CXI instrument (Boutet & Williams, 2010 ▶; Liang *et al.*, 2015 ▶) was configured in what has become a standard serial femtosecond crystallography (SFX) setup using a Gas Dynamic Virtual Nozzle (GDVN) system (DePonte *et al.*, 2008 ▶; Weierstall, 2014 ▶). The experimental geometry was the same as that first reported during the initial demonstration of the high-resolution capabilities of SFX (Boutet *et al.*, 2012 ▶), with the only difference that Kirkpatrick–Baez focusing mirrors (Siewert *et al.*, 2012 ▶) have since replaced compound refractive lenses as the X-ray focusing element.

The transmitted FEL pulses were focused to a ∼1 µm × 1 µm FWHM spot where a liquid jet carrying crystals of the model protein lysozyme was intercepted by the X-ray beam. Each FEL pulse resulted in a recorded diffraction pattern using the Cornell–SLAC Pixel Array Detector (CSPAD) (Hart *et al.*, 2012*a* ▶ *b* ▶; Blaj *et al.*, 2015 ▶). The relatively low photon energy of 7.125 keV effectively limited the achievable resolution in the SFX measurement to 2.3 Å at the edges of the CSPAD. The incident intensity available at CXI was reduced by 30% because of the diamond crystal. Furthermore, the transmission was again reduced intentionally using attenuators to prevent the most intense Bragg peaks from saturating the pixels of the CSPAD. The limited dynamic range of the CSPAD regularly forces the LCLS beam to be attenuated and this is no different in the presented case compared with non-multiplexed operations, where the incident intensity is optimized to optimally use the dynamic range of the CSPAD.

#### 2.2.2. Femtosecond XANES measurement at XPP   

The XPP instrument (Chollet *et al.*, 2015 ▶) used a standard configuration to repeat a femtosecond time-resolved XANES experiment reported earlier (Lemke *et al.*, 2013*a* ▶) on the photoexcited Spin Cross-Over (SCO) in an aqueous solution of a prototypical molecule: iron bipyridine [Fe-(bpy)_3_]^2+^. A 400 nm optical pump laser pulse with ∼50 fs pulse duration was used for photoexcitation. The optical pump and X-ray probe beam were incident nearly collinearly (within 1° on the sample). The total fluorescence yield was measured as a function of time delay between the two pulse trains using a point detector (*i.e.* a PIPS™ diode, Canberra, Inc.).

Two crucial aspects for a high-quality X-ray pump and probe measurement were the pulse-to-pulse intensity normalization and the pulse-to-pulse timing jitter correction. The intensity normalization was achieved by using a partially transmissive single-shot intensity monitor (Feng *et al.*, 2011 ▶). The timing jitter correction was achieved using a partially transmissive timing tool based on the spectral encoding method (Bionta *et al.*, 2014 ▶; Harmand *et al.*, 2013 ▶; Lemke *et al.*, 2013*b* ▶). The timing tool requires sufficient intensity in the incident X-ray beam for generating sufficient changes in the refractive index of the interaction medium for high-quality signal. As such, there were some initial concerns over whether this technique could be used with a weaker monochromatic X-ray beam. The incident X-ray beam was then focused to a typical spot of size ∼100 µm and a thicker target was also used for the timing tool to compensate for the lower monochromatic X-ray pulse energy. Clear timing signal was thus obtained for a large fraction (>50%) of the individual pulses.

Under similar FEL performances, the smaller bandwidth of the diamond 〈111〉 monochromator as opposed to the Si 〈111〉 monochromator used in the original experiment reduces the incoming X-ray flux to the time tool by an additional factor of ∼2. For the current example experiment this further reduction in the incident X-ray flux was compensated by averaging the data over approximately twice as many FEL pulses to achieve an identical signal/noise ratio to the original. The longer data acquisition time constituted a greater fraction of the total allocated beam time by roughly half a shift. To mitigate the need for increase in the total number of shifts, the experimental setup could in principle be optimized to account for the lower signal, by higher signal amplification and increasing the solid angle used for total fluorescence detection.

The slight increase in the FEL natural divergence in the diffraction plane resulted in astigmatic focusing where the vertical and horizontal foci were separated by as much as 300 mm, depending on the incidence angle on the diamond crystal. For the the experiments requiring a focal size 50 µm or greater, the effect of astigmatic focusing is negligible.

## 3. Results and discussions   

### 3.1. Structure of lysozyme:gadolinium   

**Figure 2:**
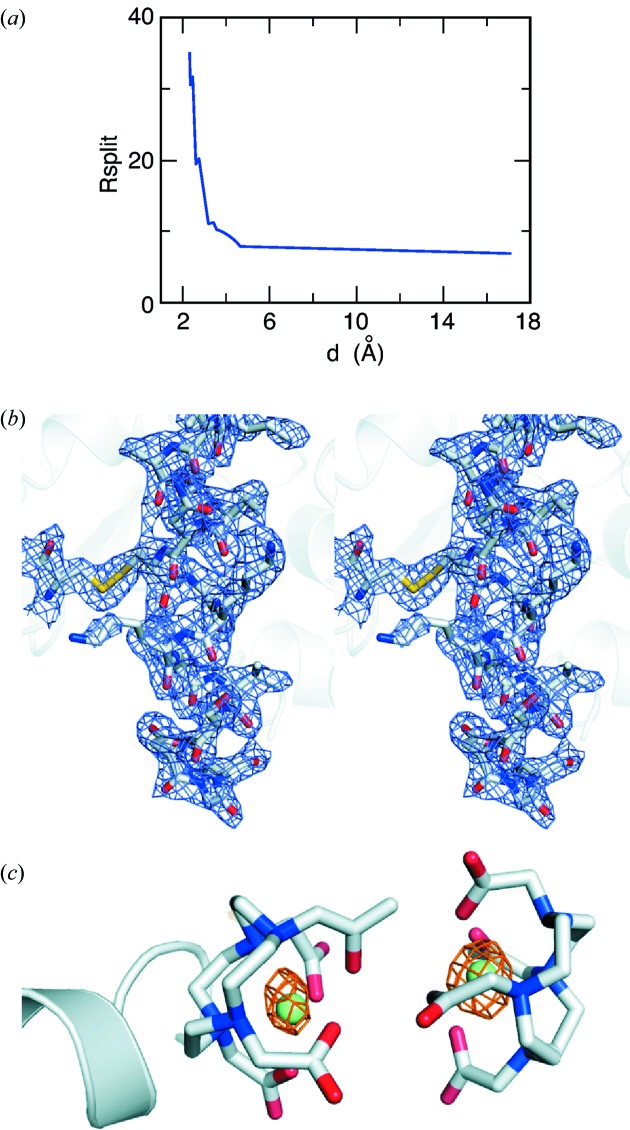
(*a*) *R*_split_ *versus* resolution. (*b*) Final refined 2*mF*_*o*_ − *DF*_*c*_ map contoured at 1σ for lysozyme:gadolinium using data collected in the multiplexing mode, overlayed on the final refined structure. (*c*) Anomalous difference density map phased with molecular replacement phases from a model without gadolinium atoms. The map (orange mesh) was contoured at 4σ.

The individual diffraction patterns from the CSPAD were processed with the *Cheetah* program for correction and hit-finding (Barty *et al.*, 2014 ▶) and then indexed, merged and integrated using *CrystFEL* (White *et al.*, 2012 ▶). Out of 203031 hits, 53661 diffraction patterns could be indexed (26.4%). The resolution limit was set at 2.3 Å based on *R*_split_ [see Fig. 2(*a*) ▶ for a plot of *R*_split_ *versus* resolution]. The overall *R*_split_ (White *et al.*, 2012 ▶) was 11.3% and the Wilson *B*-factor was 36.4 Å^2^. These values are within the typical range observed for non-multiplexed similar experiments at CXI with the observed difference likely attributable to the specifics of the sample.

The data were phased using molecular replacement with *PHASER* (McCoy, 2007 ▶; McCoy *et al.*, 2007 ▶) using native lysozyme (PDB entry 1vds). Structure determination was trivial. The rotation and translation function *Z*-scores (RFZ, TFZ) were 13.3 and 26.3, respectively. Refinement using *REFMAC5* resulted in an excellent model with an *R*-factor of 0.225 and an *R*_free_ of 0.260. Fig. 2(*b*) ▶ shows the refined 2*mF*_o_ − *DF*_*c*_ map (Read, 1986 ▶). RMS deviations from ideal geometry are 0.014 Å for bond lengths and 1.66° for bond angles. 97.6% and 2.4% of residues are in preferred and allowed regions of the Ramachandran plot, respectively.

An anomalous difference Fourier map was calculated using phases obtained directly from molecular replacement (*i.e.* without gadolinium atoms). This map contained high peaks (10 and 6σ) at the positions of the gadolinium atoms (Fig. 2*c* ▶).

The current data were compared with the lysozyme:gadolinium data previously used to demonstrate *de novo* phasing using FEL data (Barends *et al.*, 2014 ▶). These data were collected under similar circumstances without multiplexing, and using 8.4 keV photons rather than 7.1 keV. The correlation on structure factor amplitudes between the two data sets is 96.3%. No significant differences were observed between the two structures with the differences in structure factors most likely attributable to the different photon energies used, with significant differences in the anomalous signal between 8.4 and 7.1 keV. For the multiplexing data, the anomalous correlation between random half-data sets (CC_ano_) is 7.3% *versus* 48% for the 8.4 keV data, which is consistent with the much lower *f*′′ for gadolinium at the photon energy used for the multiplexing experiment (4e^−^ at 7.1 keV *versus* 12.6 e^−^ at 8.4 keV).

### 3.2. Photo-excited spin transition dynamics in [Fe-(bpy)_3_]^2+^   

**Figure 3:**
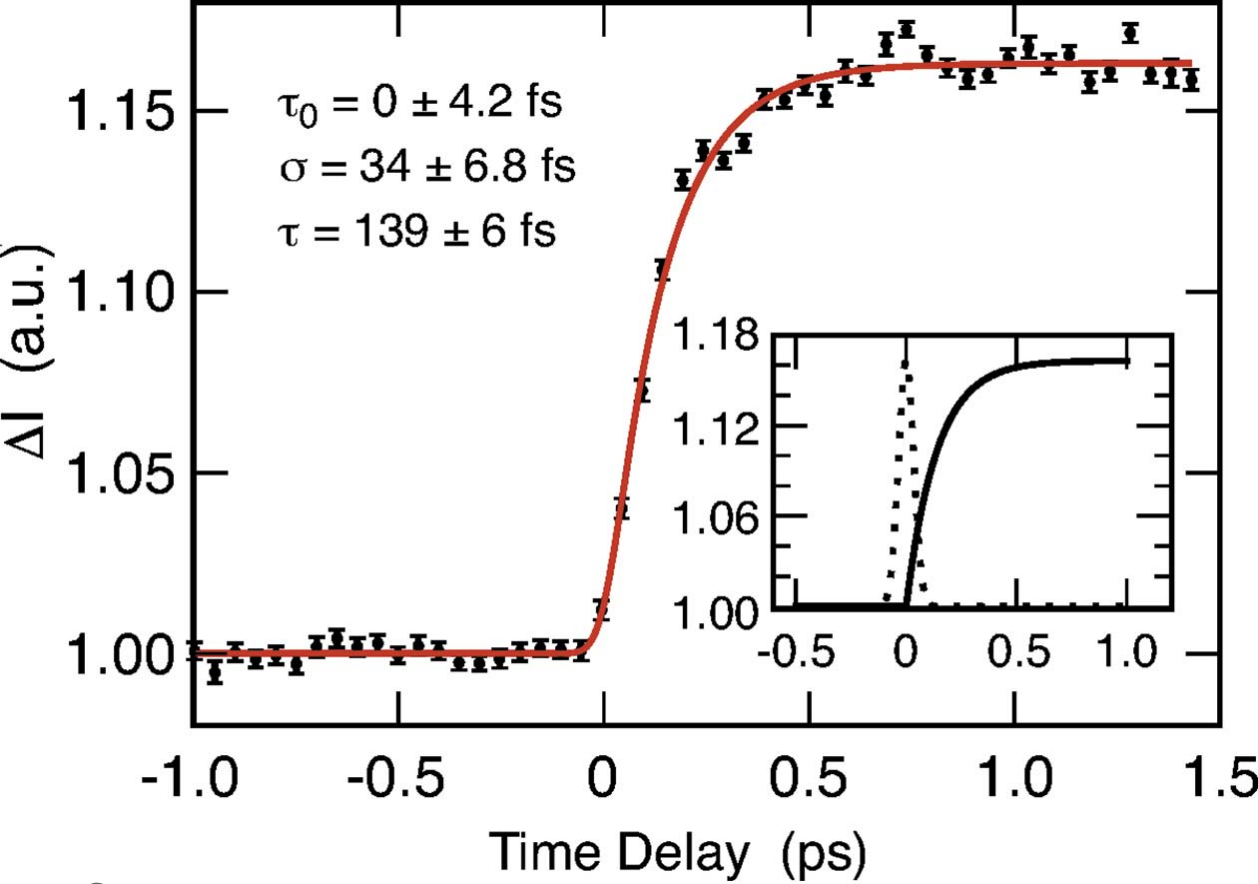
Time-dependent amplitude change of the XANES spectrum of Fe(bpy)_3_ at 7.125 keV (solid circles with error bars), characteristic for changes in the Fe–N distance in the molecule. The data were fitted by convolution of an exponential rise (with a time constant τ) with a temporal instrument response function (Gaussian). The resulting fit is shown as a red solid line. The fitting parameters are τ = 139 ± 6 fs and Gaussian’s standard deviation σ = 34 ± 6.8 fs. The inset shows the instrument response function (dashed line) and the exponential rise time (solid line) resulting from the fitting procedure.

Time-resolved XANES measurements of [Fe-(bpy)_3_]^2+^ were carried out by probing transient absorption changes of the Fe *K*-edge and thereby changes of the local geometric environment and charge distribution of the Fe atom. In the [Fe-(bpy)_3_]^2+^ molecule, the central Fe atom is surrounded in octahedral shape by the nitrogen atoms from the bipyridine (bpy) ligands. Upon photoabsorption, one electron from the Fe atom is excited to a Metal-to-Ligand Charge Transfer (MLCT) manifold, changing the oxidation state. From here, within 10–100 fs, the molecule relaxes into a High-Spin (HS) quintet state with a 0.2 Å increase in Fe—N bond length. The changed geometry changes the multiple-scattering conditions for an ionized 1*s* electron at the Fe *K*-edge which can be probed by changes in the XANES spectrum compared with the non-excited state. The largest change in the spectrum, at 7.125 keV, can be used to probe the transient population of the HS state as shown in Fig. 3 ▶.

The time dependence of the amplitude of change in the difference spectra in Fig. 3 ▶ is very similar in quality to that obtained in the first measurement in Fig. 5(*b*) of Lemke *et al.* (2013*a* ▶) and was modelled by an exponential rise convolved by the instrument resolution. The time constant τ of the exponential rise in the current measurement was determined to be 139 ± 6 fs, slightly shorter than the 163 ± 6 fs value obtained previously. The instrument resolution was, however, improved by about a factor two, thanks to improvement in the timing diagnostics (Harmand *et al.*, 2013 ▶). Furthermore, the higher energy resolution in XANES afforded by using the diamond monochromator could potentially provide valuable information in some specific cases to distinguish, for example, fine energy structure levels in the pre-edge spectrum.

The intensity reduction but improved energy resolution when operating in the beam-sharing mode did not affect the feasibility of the experiment. The time needed to acquire data at the same signal/noise level was approximately doubled. The complex motions of the large-offset DCM compared with a channel-cut monochromator used in the original measurement increased the time required to optimally align the monochromator settings when varying the X-ray energy. The energy axis could therefore be chosen to be the slower scanning axis if required as opposed to the timing variable.

## 4. Conclusions   

Multiplexing of the LCLS beam was demonstrated for hard X-rays by spectral division using a near-perfect thin diamond crystal monochromator operating in the Bragg geometry. The wavefront and coherence properties of both the reflected and transmitted beams are preserved, allowing simultaneous experiments for the determination of the structure of a model protein and the studies of the photoexcited spin crossover dynamics in a prototypical molecule. The results of both measurements were similar to those obtained separately using dedicated non-multiplexed beams with no discernible differences in data quality, validating the viability of such a scheme to be applied to more experiments.

To extend this scheme, the transmitted beam can be split one more time using a second thin diamond crystal as in the XPP–XCS–CXI beam-sharing scenario (Feng *et al.*, 2013*a* ▶) involving an additional LCLS hard X-ray instrument, the X-ray Correlation Spectroscopy (XCS) instrument (Alonso-Mori *et al.*, 2015 ▶). As such, three simultaneous experiments could be conducted, two using monochromatic beams (XPP, XCS) and one using the transmitted beam with two spectral notches (CXI). Another beam-sharing scheme using the time-sharing principle has also been demonstrated and practiced regularly. It involves the Material under Extreme Conditions (MEC) instrument (Nagler *et al.*, 2015 ▶) when it operates in a mode whereby the FEL beam is only used once every few minutes. A mirror is then used to steer the FEL to a different instrument during the MEC’s idle time.

The multitude of various multiplexing operations at LCLS has permitted additional valuable beam time to be allocated. Experiments compatible with beam-sharing operations are regularly solicited and scheduled, effectively increasing the LCLS capacity.
